# Supplementary material for: Wnt/Beta-catenin/Esrrb signalling controls the tissue-scale reorganization and maintenance of the pluripotent lineage during murine embryonic diapause
Source: Nat Commun. 2020 Oct 30;11:5499. doi: 10.1038/s41467-020-19353-0 (PMC7603494; doi:10.1038/s41467-020-19353-0)
Supplement: Supplementary file 1 — Supplementary information [file 41467_2020_19353_MOESM1_ESM.pdf]

Supplementary Information

Wnt/Beta-catenin/Esrrb signalling controls the tissue-scale reorganization and maintenance of the pluripotent lineage during murine embryonic diapause

Fan et al

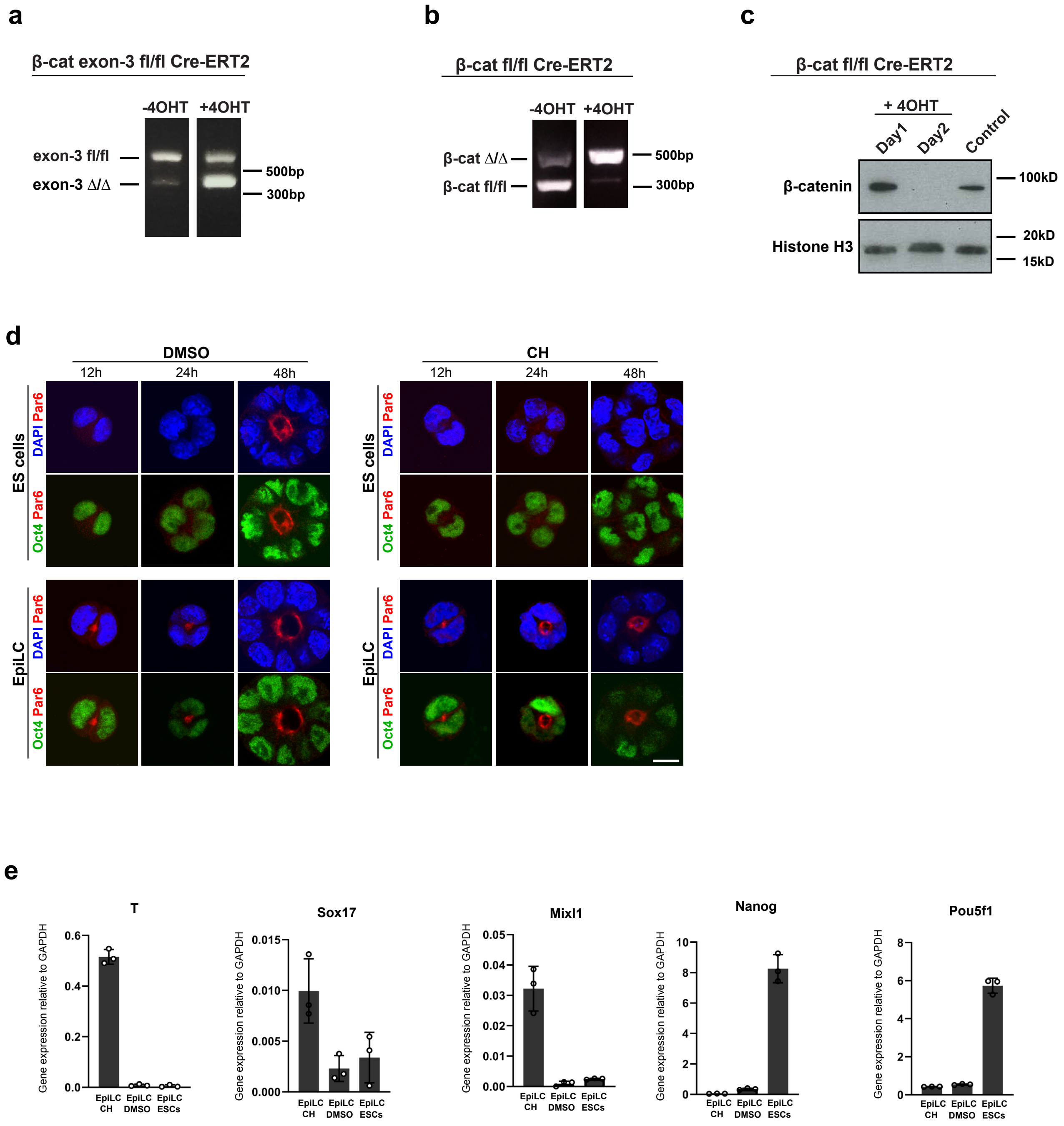

Supplementary Figure 1. Active Wnt/b-catenin signalling blocks the establishment of epithelial polarity in a 3D in vitro model of epiblast development. (a) PCR analysis of b-catenin exon-3 deletion after 2 days of 4OHT treatment of b-catenin exon-3 fl/fl Cre-ERT2 ES cells, compared to non-treated (-4OHT) control cells. (b) PCR analysis of conditional b-catenin deletion after 2 days of 4OHT treatment of b-catenin fl/fl Cre-ERT2 ES cells, compared to non-treated (-4OHT) control cells. (c) Western blot analysis of b-catenin protein levels after 1 and 2 days of 4OHT treatment of b-catenin fl/fl Cre-ERT2 ES cells, compared to non-treated (-4OHT) control cells. Uncropped blots are provided in source data file. (d) EpiLC or ES cells cultured in the presence of DMSO or CH for 12h, 24h or 48 h and stained for Par6, Oct4 and DAPI. Scale bar, 10 µm. (e) Gene expression of mesendodermal (T, Sox17 and Mixl1) and pluripotency (Oct4 and Nanog) markers, relative to GAPDH, in EpiLC treated with CH or DMSO for 48h and ES cells grown in 2i/Lif. Data represents mean ± SD, three independent experiments. Related to Figure 2 and Figure 3.

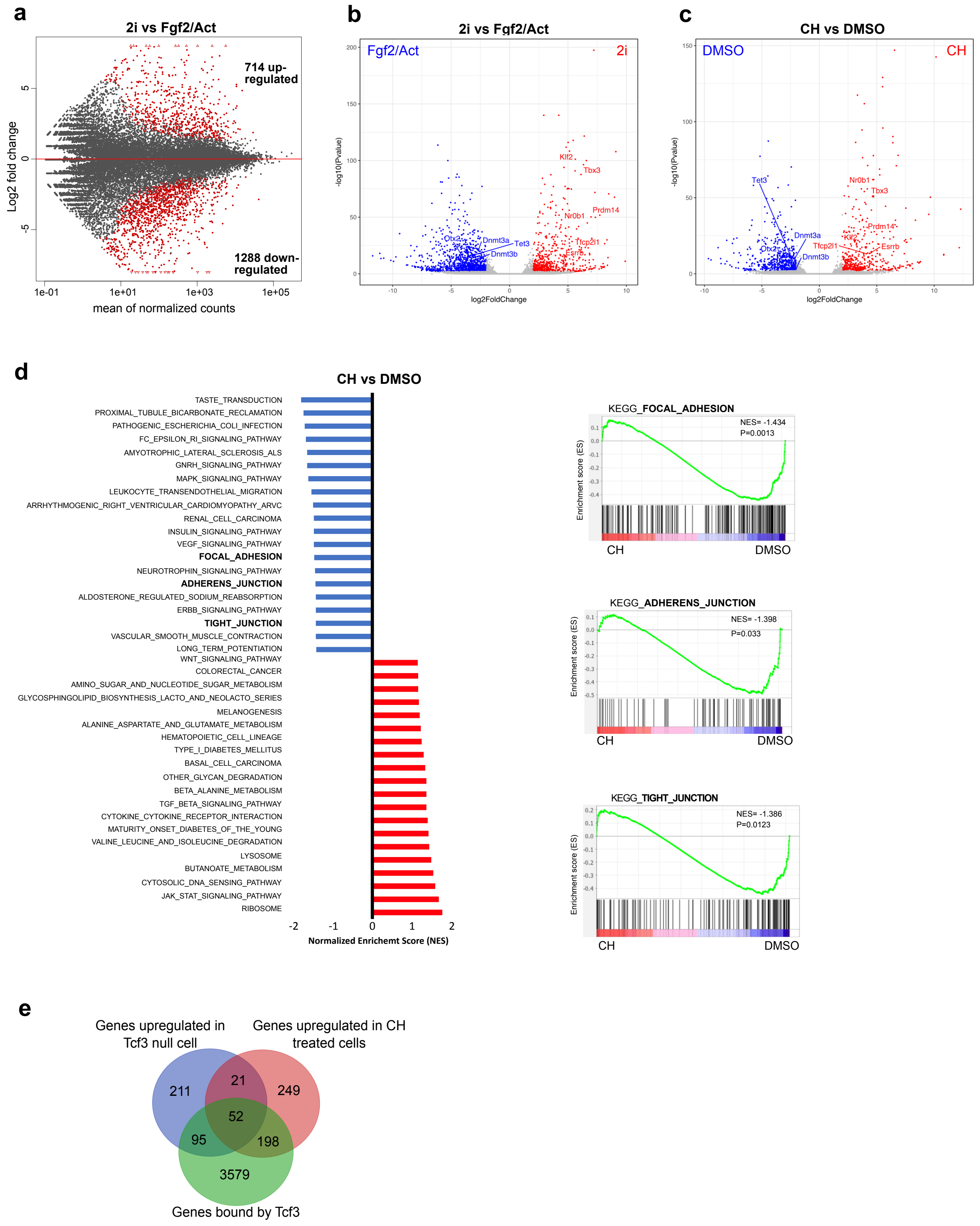

Supplementary Figure 2. Wnt signalling counters epithelialization in the context of naïve pluripotency. (a) Scatter plot of gene expression of 2i and Fgf2/Activin treated cells with differentially expressed genes that are significantly up- or downregulated shown in red, adjusted p-value < 0.01, three replicates per culture condition. (b) Volcano plot of gene expression of 2i and Fgf2/Activin treated cells with differentially expressed genes that are significantly upregulated in 2i shown in red and in Fgf2/Activin shown in blue, adjusted p-value < 0.01, three replicates per culture condition. (c) Volcano plot of gene expression of CH and DMSO treated cells with differentially expressed genes that are significantly upregulated in CH shown in red and in DMSO shown in blue, adjusted p-value < 0.01, three replicates per culture condition. (d) GSEA plots showing enrichment of Focal adhesion, Adherens and Tight junction KEGG pathways in DMSO treated cells in comparison to CH treated ES cells. (e) Putative Wnt targets identified via intersecting genes upregulated upon CH treatment, Tcf3 depletion and bound by Tcf3. Related to Figure 3.

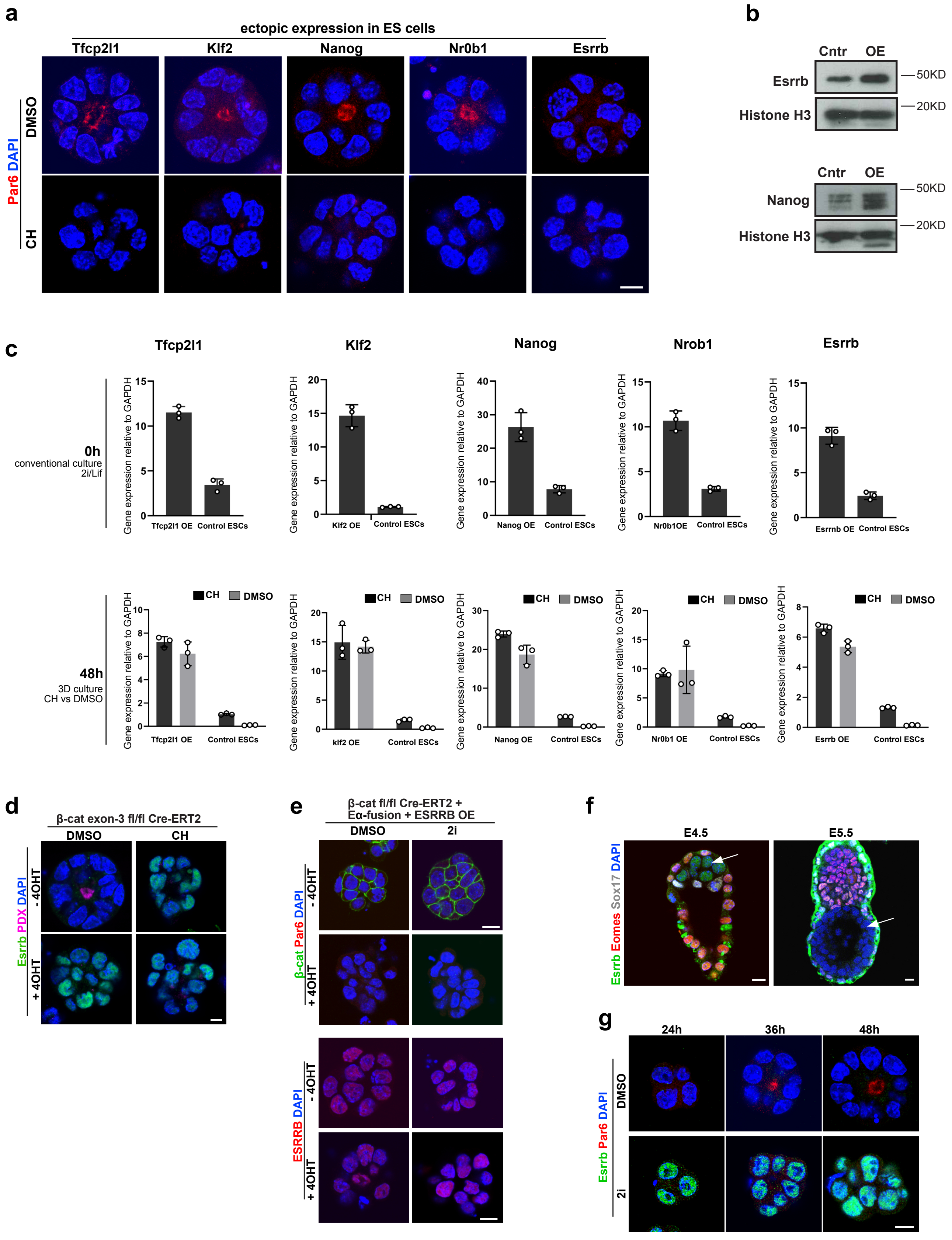

Supplementary Figure 3. Wnt signalling supresses epithelialization via Esrrb. (a) E14 ES cells expressing ectopically Tfcp2l1, Klf2, Nanog, Nr0b1 or Esrrb transgenes, cultured in Matrigel for 48 h in the presence of DMSO or CH and stained for Par6 and DAPI. (b) Western blot analysis of Esrrb and Nanog expression in E14 (control) and over expressing (OE) cell lines cultured in 2i/Lif supplemented medium. Histone H3 serves as loading control. Uncropped blots are provided in source data file. (c) Gene expression of Tfcp2l1, Klf2, Nanog, Nr0b1 and Esrrb relative to GAPDH, compared to control wild-type ES cells grown in conventional 2i/Lif culture conditions (0h) and at 48h of 3D culture in CH or DMSO supplemented medium. Data represents mean ± SD, three independent experiments. (d) Esrrb expression and establishment of epithelial polarity in control (-4OHT) b-catenin exon-3 fl/fl and b-catenin exon-3 Δ/Δ (+4OHT) ES cells cultured in the presence of CH or DMSO for 48h in 3D culture conditions. (e) Conditional ablation of b-catenin in b-catenin fl/fl Cre-ERT2 ES cells ectopically expressing E-cadherin-α-catenin fusion (Eα-fusion) and Esrrb. The cells were exposed to 4OHT for 2 days and after that cultured for 48 h in 3D culture conditions, in the presence of DMSO or 2i and stained for b-catenin, Par6 and DAPI, upper panel and Esrrb and DAPI, lower panel. See also Figures 2g and 2j. (f) E4.5 blastocyst and E5.5 egg cylinder stage embryos stained for Esrrb, Eomes and Sox17. Arrows indicate the pre- and post-implantation epiblast at E4.5 and E5.5, respectively. The nuclei are counterstained with DAPI. (g) Wild-type E14 ES cells grown in 3D culture conditions in the presence of DMSO or 2i for 24 h, 36 h and 48 h and stained for Par6, Esrrb and DAPI. Scale bars, 10 μm. Related to Figure 3.

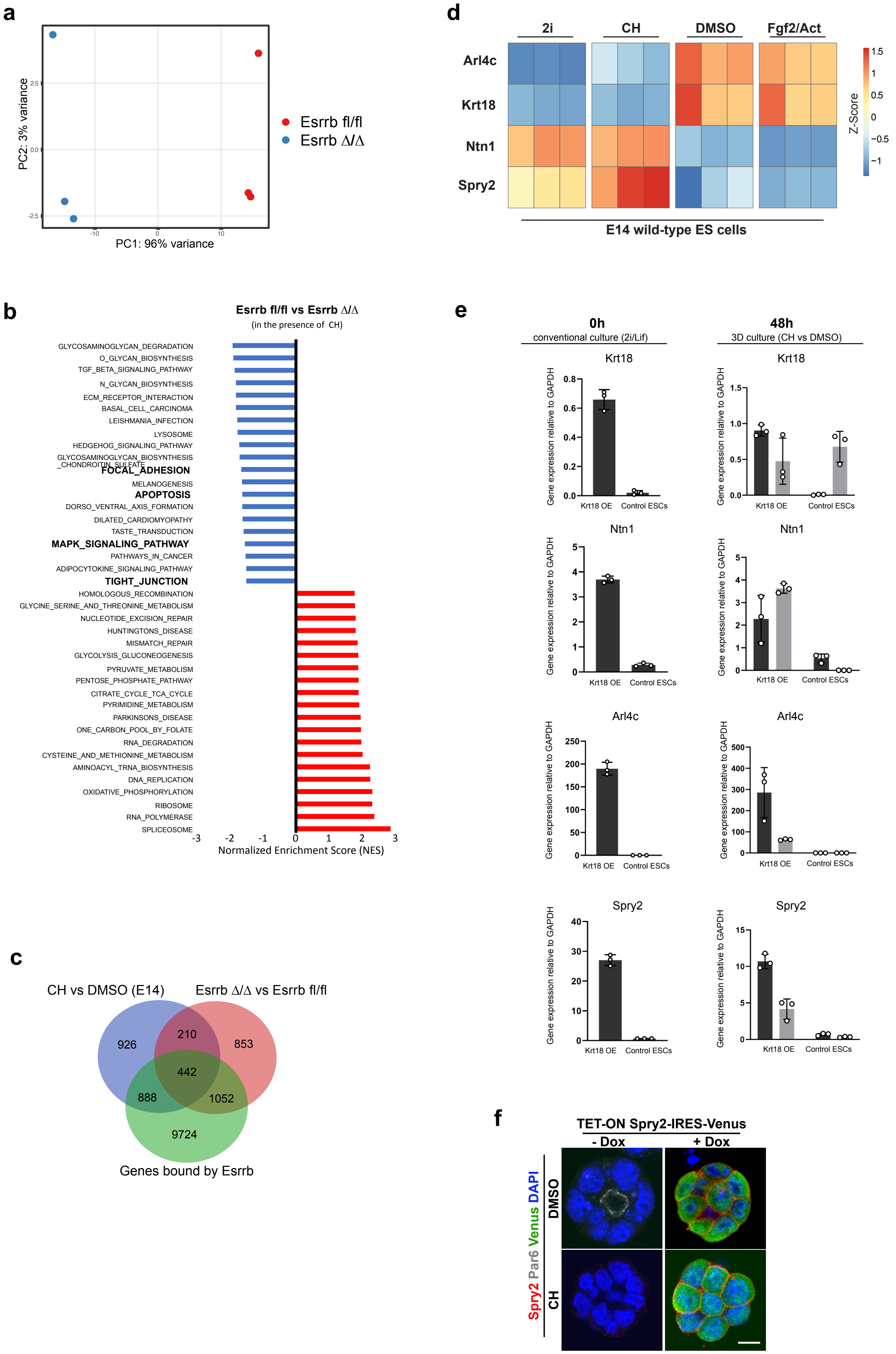
a
PC2: 3% variance
PC1: 96% variance
Esrrb fl/fl
Esrrb Δ/Δ
b
Esrrb fl/fl vs Esrrb Δ/Δ
(in the presence of CH)
GLYCOSAMINOGLYCAN_DEGRADATION
O_GLYCAN_BIOSYNTHESIS
TGF_BETA_SIGNALING_PATHWAY
N_GLYCAN_BIOSYNTHESIS
ECM_RECEPTOR_INTERACTION
BASAL_CELL_CARCINOMA
LEISHMANIA_INFECTION
LYSOSOME
HEDGEHOG_SIGNALING_PATHWAY
GLYCOSAMINOGLYCAN_BIOSYNTHESIS_CHONDROITIN_SULFATE
FOCAL_ADHESION
MELANOGENESIS
APOPTOSIS
DORSO_VENTRAL_AXIS_FORMATION
DILATED_CARDIOMYOPATHY
TASTE_TRANSDUCTION
MAPK_SIGNALING_PATHWAY
PATHWAYS_IN_CANCER
ADIPOCYTOKINE_SIGNALING_PATHWAY
TIGHT_JUNCTION
HOMOLOGOUS_RECOMBINATION
GLYCINE_SERINE_AND_THREONINE_METABOLISM
NUCLEOTIDE_EXCISION_REPAIR
HUNTINGTONS_DISEASE
MISMATCH_REPAIR
GLYCOLYSIS_GLUCONEOGENESIS
PYRUVATE_METABOLISM
PENTOSE_PHOSPHATE_PATHWAY
CITRATE_CYCLE_TCA_CYCLE
PYRIMIDINE_METABOLISM
PARKINSONS_DISEASE
ONE_CARBON_POOL_BY_FOLATE
RNA_DEGRADATION
CYSTEINE_AND_METHIONINE_METABOLISM
AMINOACYL_TRNA_BIOSYNTHESIS
DNA_REPLICATION
OXIDATIVE_PHOSPHORYLATION
RIBOSOME
RNA_POLYMERASE
SPLICEOSOME
Normalized Enrichment Score (NES)
c
CH vs DMSO (E14)
Esrrb Δ/Δ vs Esrrb fl/fl
926
210
853
442
888
1052
9724
Genes bound by Esrrb
d
2i
CH
DMSO
Fgf2/Act
Arl4c
Krt18
Ntn1
Spry2
Z-Score
E14 wild-type ES cells
e
0h
conventional culture (2i/Lif)
48h
3D culture (CH vs DMSO)
Krt18
Ntn1
Arl4c
Spry2
Gene expression relative to GAPDH
Krt18 OE
Control ESCs
f
TET-ON Spry2-IRES-Venus
- Dox
+ Dox
DMSO
CH
Spry2 Par6 Venus DAPI

Supplementary Figure 4. Esrrb controls the epithelial program via Spry2. (a) Principal component analysis (PCA) plot of RNA-seq datasets of Esrrb Δ/Δ and Esrrb fl/fl cells cultured in CH supplemented medium for 24 h. (b) GSEA plots showing enrichment of Focal adhesion, Tight junction, Apoptosis and MAPK signalling KEGG pathways in Esrrb Δ/Δ cells compared to Esrrb fl/fl cells. (c) Putative Esrrb targets identified via intersecting genes modulating their expression upon CH / DMSO treatment in wild type ES cells, Esrrb depletion and bound by Esrrb. The differentially expressed genes from CH / DMSO treatment in wild type ES cells and Esrrb depletion have at least 2-fold change in expression and $p<0.02$ (two-tailed unpaired Student's t test). (d) Gene expression of epithelial factors in wild-type E14 ES cells cultured in DMSO, 2i, CH or Fgf2/Activin supplemented medium. (e) Gene expression of Krt18, Ntn1, Arl4c and Spry2 relative to GAPDH, compared to control wild-type ES cells in conventional 2i/Lif culture conditions (0h) and at 48h of 3D culture in CH or DMSO supplemented medium. Data represents mean ± SD, three independent experiments. (f) Ectopic expression of Spry2 in E14 ES cells (+Dox), compared to unstimulated (-Dox) isogenic control. The cells were cultured for 48 h in the presence of DMSO or CH and stained for Spry2, Par6, Venus and DAPI. Scale bar, 10 µm. Related to Figure 4.

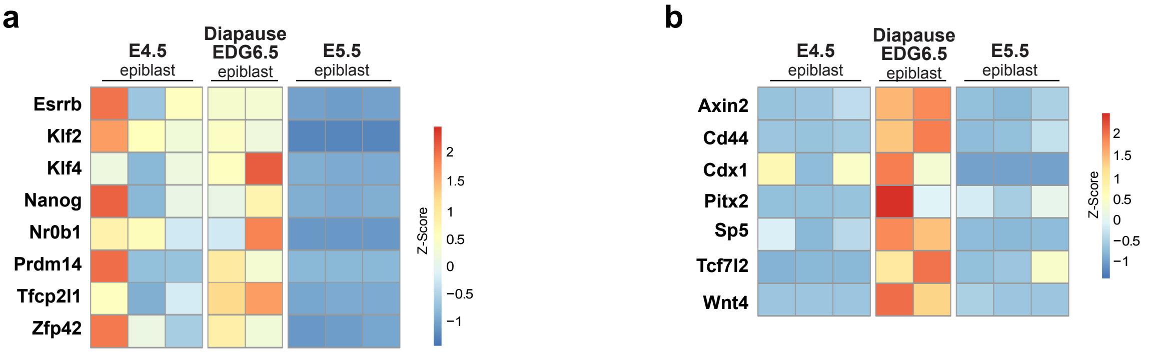

Supplementary Figure 5. Dynamics of Wnt signalling activity during diapause. (a) Gene expression level of naïve pluripotency factors in the epiblast of E4.5, diapause (EDG6.5) and E5.5 embryos. (b) Gene expression level of Wnt signalling related factors in the epiblast of E4.5, diapause (EDG6.5) and E5.5 embryos. Related to Figure 5.

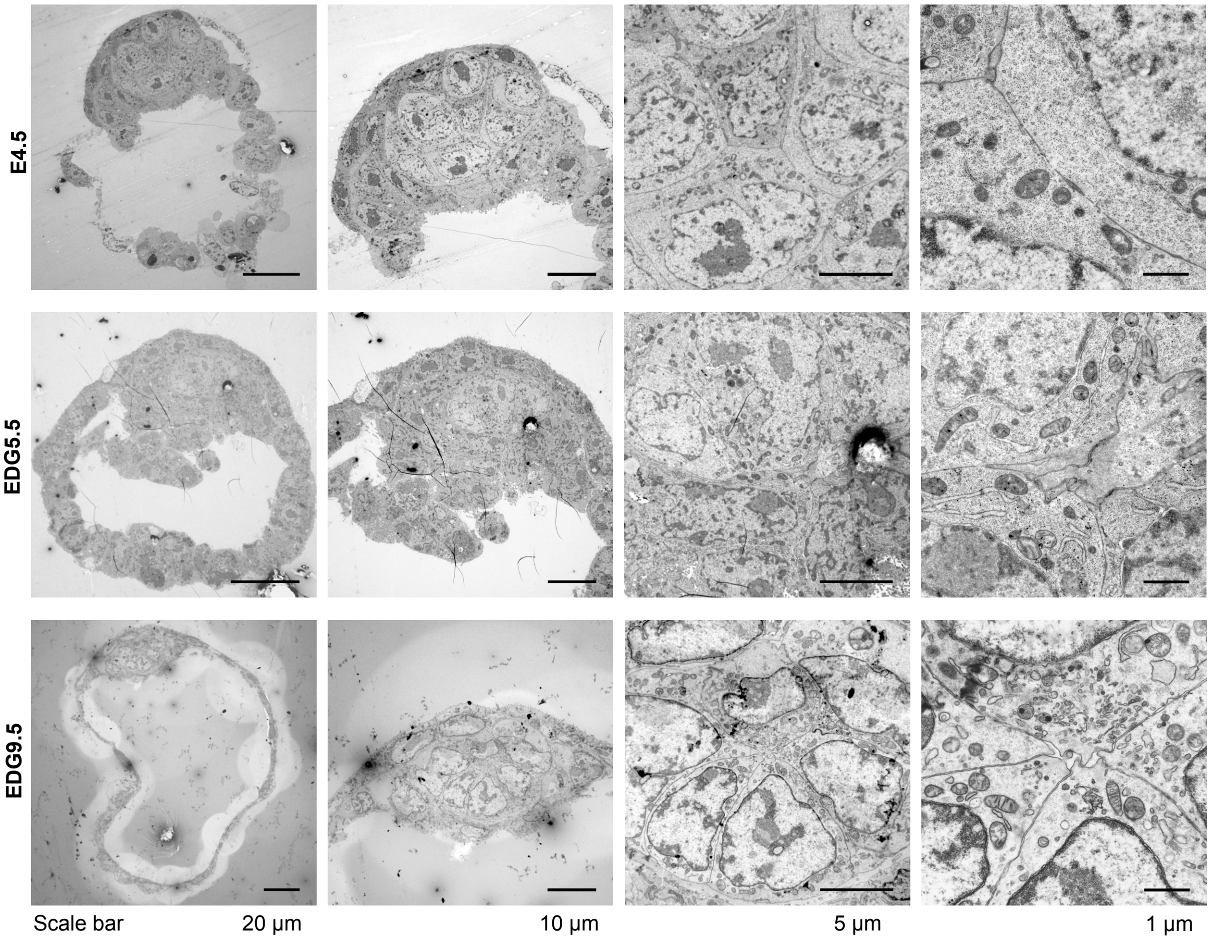

Supplementary Figure 6. Electron microscopy analysis. Electron microscopy analysis of E4.5, EDG5.5 and EDG9.5 blastocysts. Related to Figure 5.

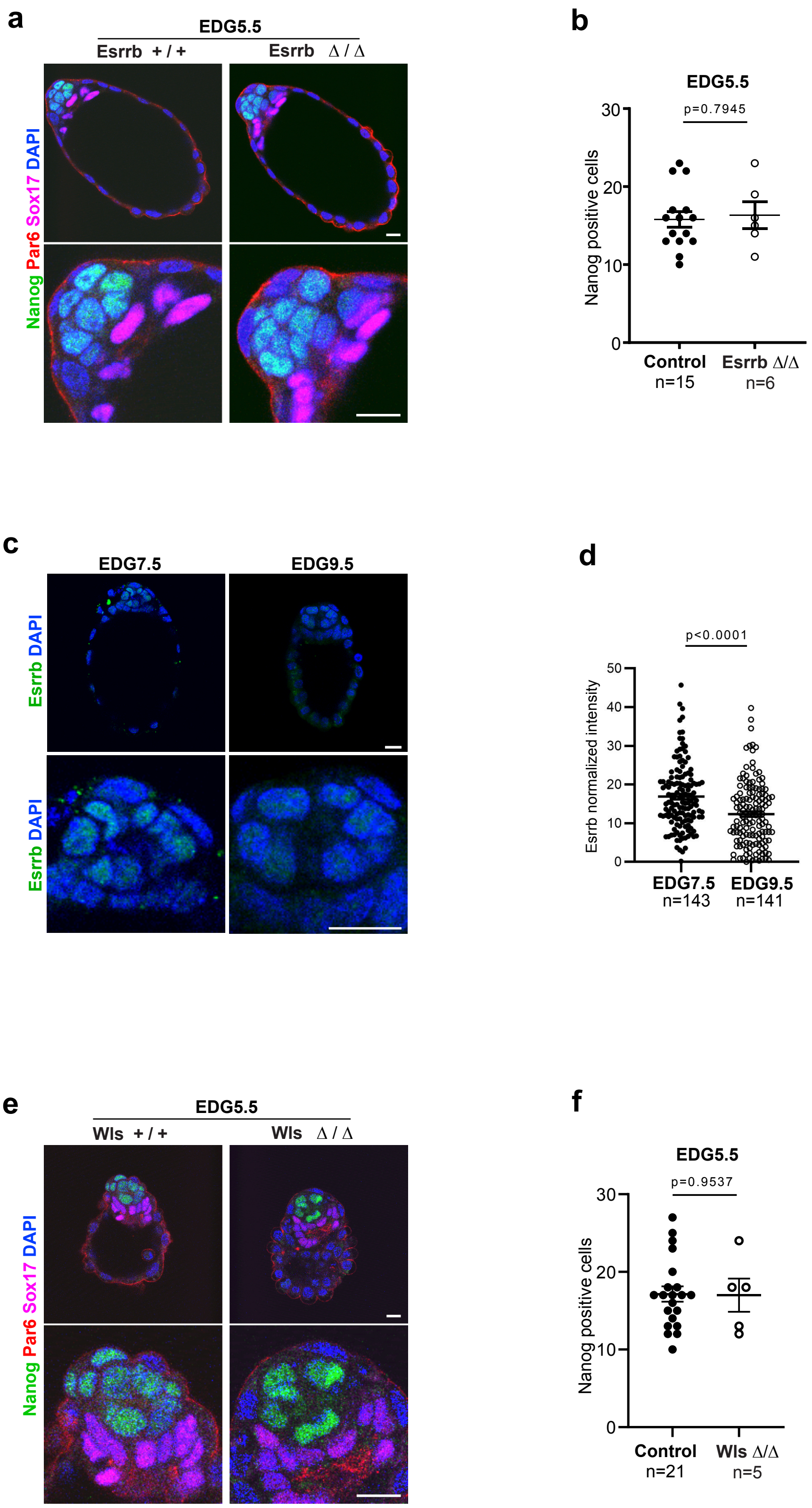

Supplementary Figure 7. Loss of function analysis of Esrrb and Wls during diapause. (a) Esrrb control (+/+) and knock-out (Δ/Δ) embryos isolated at EDG5.5 and stained for Nanog, Par6, Sox17 and DAPI. (b) Number of epiblast cells in Esrrb control (+/+ and +/Δ) and knock-out embryos (Δ/Δ) at EDG5.5 based on Nanog expression. Data represents mean ± SEM, n = number of embryos, two-tailed unpaired Student's t test, exact p-value is noted in this figure. (c) EDG7.5 and EDG9.5 diapause embryos stained for Esrrb and DAPI. (d) Esrrb expression in the epiblast cells of EDG7.5 and EDG9.5 diapause embryos. Data represents mean ± SEM, n = cells measured in 8 EDG7.5 and 10 EDG9.5 embryos, two-tailed unpaired Student's t test, exact p-value is noted in this figure. (e) Wls control (+/+) and knock-out (Δ/Δ) embryos isolated at EDG5.5 and stained for Nanog, Par6, Sox17 and DAPI). (f) Number of epiblast cells in Wls control (+/+ and +/Δ) and knock-out embryos (Δ/Δ) at EDG5.5 based on Nanog expression. Data represents mean ± SEM, n = number of embryos, two-tailed unpaired Student's t test, exact p-value is noted in this figure. Scale bars, 10 µm. Related to Figures 6, 7 and 8.

Supplementary Table 1. Primers used in this study.

Name	Sequence	Purpose	PCR products
ESRRB_F	CAGCTTCCACTTTTTGTGAGG	Genotyping	WT band 213bp; KO band 351bp; Floxed band 255bp;
ESRRB_R1	ACGGGTATGACGTAGGAGAGC	Genotyping	
ESRRB_R2	TAGGCTTTTGGAGGGAGGGTTCAT	Genotyping	
H2BGFP_F	AAGGCCGTCACCAAGTACAC	Genotyping	H2B-GFP band 300bp
H2BGFP_R	AAGTCGTGCTGCTTCATGTG	Genotyping	
Wls_lox_F	AGGCTTCGAACGTAACTGACC	Genotyping	WT band 411 bp; Floxed band 556 bp
Wls_lox_R	CTCAGAACTCCCTTCTTGAAGC	Genotyping	
Wls_lox_F2	CTTCCCTGCTTCTTTAAGCGTC	Genotyping	WT band 1625 bp; KO band 410 bp
Wls_lox_R2	CTCAGAACTCCCTTCTTGAAGC	Genotyping	
Beta-Cat_Ko_F	AAT CAC AGG GAC TTC CAT ACC AG	Genotyping	KO band 631 bp
Beta-Cat_Ko_R	GCC CAG CCT TAG CCC AAC	Genotyping	
Beta-Cat_Flox_F	AAG GTA GAG TGA TGA AAG TTG TT	Genotyping	KO band 500 bp; Flox band 324 bp; WT band 221 bp
Beta-Cat_Flox_R	CAC CAT GTC CTC TGT CTA TTC	Genotyping	
Beta-Cat_Flox_F1	TAC ACT ATT GAA TCA CAG GGA CTT	Genotyping	
b-cat Ex2S_Exon3_F	GACACCGCTGCGTGGACAATG	Genotyping	Floxed band 700 bp; Delta band 400 bp; WT band 500 bp
b-cat Ex3AS_Exon3_R	GTGGCTGACAGCAGCTTTTCTG	Genotyping	
Cre_F	CAA GTT GAA TAA CCG GAA ATG	Genotyping	Cre band 800 bp
Cre_R	GCC AGG TAT CTC TGA CCA GA	Genotyping	
Nanog_F	AGGGTCTGCTACTGAGATGCTCTG	qRT-PCR	
Nanog_R	CAACCACTGGTTTTTCTGCCACCG	qRT-PCR	
Hprt_F	ATGAGCGCAAGTTGAATCTG	qRT-PCR	
Hprt_R	CAGATGGCCACAGGACTAGA	qRT-PCR	
Gapdh_F	TGAAGCAGGCATCTGAGGG	qRT-PCR	
Gapdh_R	CGAAGGTGGAAGAGTGGGAG	qRT-PCR	
Oct4_F	AGACCATGTTTCTGAAGTGCCCG	qRT-PCR	
Oct4_R	CGCCGGTTACAGAACCATACTCG	qRT-PCR	
T_F	TTGAACTTTCCTCCATGTGCTGA	qRT-PCR	
T_R	TCCCAAGAGCCTGCCACTTT	qRT-PCR	
Klf2_F	ACCAAGAGCTCGCACCTAAA	qRT-PCR	
Klf2_R	GTGGCACTGAAAGGGTCTGT	qRT-PCR	
Nr0b1_F	CCAGGCCATCAAGAGTTTCT	qRT-PCR	
Nr0b1_R	CCCTCAATGTATTTCACGCA	qRT-PCR	
TFCP2L1_F	GGGGACTACTCGGAGCATCT	qRT-PCR	
TFCP2L1_R	TTCCGATCAGCTCCCTTG	qRT-PCR	
Esrrb_F	ACATTGCCTCTGGCTACCAC	qRT-PCR	
Esrrb_R	CGATGTTGCCTTGAATGGTT	qRT-PCR	
Sox17_F	GCTAGGCAAGTCTTGGAAGG	qRT-PCR	
Sox17_R	CTTGTAGTTGGGGTGGTCCT	qRT-PCR	
Spry2_F	GGTCTCGGAGCAGTACAAGG	qRT-PCR	
Spry2_R	CTCCGATTTAGGCTGCACTC	qRT-PCR	
Arl4c_F	AGTCTCTGCACATCGTTATGC	qRT-PCR	
Arl4c_R	GGTGTTGAAGCCGATAGTGGG	qRT-PCR	
Ntn1_F	TCCAAAGGCAAGCTGAAGAT	qRT-PCR	
Ntn1_R	ACGGTGAACTTCCACCAGTC	qRT-PCR	
Krt18_F	AGATTGCCAGCTCTGGATTG	qRT-PCR	
Krt18_R	TGGTGACAACTGTGGTACTC	qRT-PCR	
