# Supplementary material for: Wnt/Beta-catenin/Esrrb signalling controls the tissue-scale reorganization and maintenance of the pluripotent lineage during murine embryonic diapause
Source: Nat Commun. 2020 Oct 30;11:5499. doi: 10.1038/s41467-020-19353-0 (PMC7603494; doi:10.1038/s41467-020-19353-0)
Supplement: Supplementary file 3 — Description of Additional Supplementary Files [file 41467_2020_19353_MOESM3_ESM.pdf]

Title: Supplementary Data 1.

Description: Gene expression levels in wild-type E14 ES cells grown in 3D culture for 48 h in the presence of CH or DMSO. Genes were considered as deregulated if |log2FC|> 1 and FDR < 0.01 using Benjamini-Hochberge multiple test adjustment performed by DEseq2. Related to Figures 3 and Supplementary Fig. 2.

Title: Supplementary Data 2.

Description: Gene expression levels in wild-type E14 ES cells grown in 3D culture for 48 h in the presence of 2i or Fgf2/Activin. Genes were considered as deregulated if |log2FC|> 1 and FDR < 0.01 using Benjamini-Hochberge multiple test adjustment performed by DEseq2. Related to Figures 3 and Supplementary Fig. 2.

Title: Supplementary Data 3.

Description: List of putative Wnt targets identified via intersecting genes upregulated upon CH treatment, Tcf3 depletion and bound by Tcf3. Related to Figure Supplementary Fig. 2e.

Title: Supplementary Data 4.

Description: Gene expression levels in Esrrb deleted and floxed ES cells grown in 3D culture for 24 h in the presence of CH. Genes were considered as deregulated if |log2FC|> 1 and FDR < 0.01 using Benjamini-Hochberge multiple test adjustment performed by DEseq2. Related to Figures 4 and Supplementary Fig. 4.

Title: Supplementary Data 5.

Description: List of putative Esrrb targets identified via intersecting genes modulating their expression upon CH / DMSO treatment in wild type ES cells, Esrrb depletion and bound by Esrrb. Two-tailed unpaired Student’s t test. Related to Figure Supplementary Fig. 4c.
