# Supplementary material for: Wnt/Beta-catenin/Esrrb signalling controls the tissue-scale reorganization and maintenance of the pluripotent lineage during murine embryonic diapause
Source: Nat Commun. 2020 Oct 30;11:5499. doi: 10.1038/s41467-020-19353-0 (PMC7603494; doi:10.1038/s41467-020-19353-0)
Supplement: Supplementary file 9 — Reporting Summary [file 41467_2020_19353_MOESM9_ESM.pdf]

nature research

Corresponding author(s): Ivan Bedzhov

Last updated by author(s): Oct 2, 2020

Reporting Summary

Nature Research wishes to improve the reproducibility of the work that we publish. This form provides structure for consistency and transparency in reporting. For further information on Nature Research policies, see our Editorial Policies and the Editorial Policy Checklist.

Statistics

For all statistical analyses, confirm that the following items are present in the figure legend, table legend, main text, or Methods section.

n/a	Confirmed	
☐	☒	The exact sample size (n) for each experimental group/condition, given as a discrete number and unit of measurement
☐	☒	A statement on whether measurements were taken from distinct samples or whether the same sample was measured repeatedly
☐	☒	The statistical test(s) used AND whether they are one- or two-sided<br>*Only common tests should be described solely by name; describe more complex techniques in the Methods section.*
☒	☐	A description of all covariates tested
☒	☐	A description of any assumptions or corrections, such as tests of normality and adjustment for multiple comparisons
☐	☒	A full description of the statistical parameters including central tendency (e.g. means) or other basic estimates (e.g. regression coefficient) AND variation (e.g. standard deviation) or associated estimates of uncertainty (e.g. confidence intervals)
☐	☒	For null hypothesis testing, the test statistic (e.g. F, t, r) with confidence intervals, effect sizes, degrees of freedom and P value noted<br>*Give P values as exact values whenever suitable.*
☒	☐	For Bayesian analysis, information on the choice of priors and Markov chain Monte Carlo settings
☒	☐	For hierarchical and complex designs, identification of the appropriate level for tests and full reporting of outcomes
☒	☐	Estimates of effect sizes (e.g. Cohen's d, Pearson's r), indicating how they were calculated

Our web collection on statistics for biologists contains articles on many of the points above.

Software and code

Policy information about availability of computer code

Data collection	FACS Diva for FACSAria IIu sorter; LAS software for Leica TCS-SP5 and Leica TCS-SP8; Quantstudio 3 qPCR Data Analysis Software.
Data analysis	SnapGene GSL Biotech https://www.snapgene.com/;<br>Fiji https://fiji.sc/;<br>Tophat2 http://ccb.jhu.edu/software/tophat/ index.shtml;<br>STAR https://github.com/alexdobin/STAR;<br>DESeq2 https://bioconductor.org/packages/ release/bioc/html/DESeq2.html;<br>MACS2 http://liulab.dfci.harvard.edu/MACS/;<br>IGV http://www.broadinstitute.org/igv/;<br>HTSeq http://htseq.readthedocs.io/en/ release_0.9.1/;<br>GSEA http://www.broadinstitute.org/gsea/; RRID:SCR_003199<br>Affinity Designer https://affinity.serif.com/en-gb/<br>Graphpad Prism8 https://www.graphpad.com/scientific-software/prism/

For manuscripts utilizing custom algorithms or software that are central to the research but not yet described in published literature, software must be made available to editors and reviewers. We strongly encourage code deposition in a community repository (e.g. GitHub). See the Nature Research guidelines for submitting code & software for further information.

Data

Policy information about availability of data

All manuscripts must include a data availability statement. This statement should provide the following information, where applicable:

- Accession codes, unique identifiers, or web links for publicly available datasets
- A list of figures that have associated raw data
- A description of any restrictions on data availability

All RNA sequencing data is deposited to NCBI GEO database with GEO accession number: GSE141773 [https://www-ncbi-nlm-nih-gov.ezproxy.u-pec.fr/geo/query/acc.cgi?acc=GSE141773]
The source data for Figs.1b, 2b, 2e, 2f, 3c, 3i, 3l, 4c, 4i, 5d, 5f, 6c, 6d, 6e, 7c, 7d, 7f, 8b, 8d, 8f and Supplementary Figs. S1e, S2e, S3c, S4c, S4e, S7b, S7d and S7f are provided as a Source Data file.
There is no restriction on data availability.

Field-specific reporting

Please select the one below that is the best fit for your research. If you are not sure, read the appropriate sections before making your selection.

- [x] Life sciences
- [ ] Behavioural & social sciences
- [ ] Ecological, evolutionary & environmental sciences

For a reference copy of the document with all sections, see nature.com/documents/nr-reporting-summary-flat.pdf

Life sciences study design

All studies must disclose on these points even when the disclosure is negative.

Sample size	For RNA-sequencing analysis, we analyzed 3 samples for each culture condition. The embryos we used in each experiment ranging from 4 to 43 and the cells we measured in each experiment ranging from 36 to 143. The exact numbers of the embryos we used and the cells we measured were given in each figure. No statistical method was used to determine sample size.
Data exclusions	No data were excluded from the analysis.
Replication	All experiments were performed at least three times.
Randomization	Not applicable.
Blinding	Not applicable.

Reporting for specific materials, systems and methods

We require information from authors about some types of materials, experimental systems and methods used in many studies. Here, indicate whether each material, system or method listed is relevant to your study. If you are not sure if a list item applies to your research, read the appropriate section before selecting a response.

Materials & experimental systems

n/a	Involved in the study	
[ ]	[x]	Antibodies
[ ]	[x]	Eukaryotic cell lines
[x]	[ ]	Palaeontology and archaeology
[ ]	[x]	Animals and other organisms
[x]	[ ]	Human research participants
[x]	[ ]	Clinical data
[x]	[ ]	Dual use research of concern

Methods

n/a	Involved in the study	
[x]	[ ]	ChIP-seq
[ ]	[x]	Flow cytometry
[x]	[ ]	MRI-based neuroimaging

Antibodies

Antibodies used	Mouse monoclonal anti-Esrrb R&D systems Cat# PP-H6705-00; RRID: AB_1964232<br>Mouse monoclonal anti-Nanog Cell signaling technology Cat# 8822; RRID:AB_11217637<br>Goat polyclonal anti-Sox17 R&D Cat# AF1924, RRID:AB_355060<br>Mouse monoclonal anti-Oct4 Cell signaling technology Cat# 83932, RRID:AB_2721046<br>Rabbit polycolonal Anti-Pard6B1 Santa Cruz Biotechnology Cat# sc-67393, RRID:AB_2267889<br>Mouse monoclonal Anti-Pard6B1 Santa Cruz Biotechnology Cat# sc-166405, RRID:AB_2267890

Rabbit monoclonal anti-Sox2 Cell signaling technology Cat# 23064, RRID:AB_2714146
Mouse monoclonal Anti-E-Cadherin BD Biosciences Cat# 610182, RRID:AB_397581
Goat polyclonal Anti-GFP R&D Cat# AF4240, RRID:AB_884445
Rabbit polyclonal anti-Eomes Abcam Cat# ab23345, RRID:AB_778267
Anti-beta-Catenin BD Biosciences Cat# 610154, RRID:AB_397555
Rat monoclonal Anti-Podocalyxin R&D Cat# MAB1556, RRID:AB_2166010
Rabbit monoclonal Cleaved Caspase-3 Cell Signaling Technology Cat# 9664, RRID:AB_2070042
Rabbit polyclonal Spry2 Thermo Cat#PA5-98172, RRID:AB_2610256

Validation

The primary antibodies in this study are previously validated and all relevant information can be found in the Resource Identification Portal using the respective RRID number

Eukaryotic cell lines

Policy information about cell lines

Cell line source(s)

Mouse: EpiSC_E3: Tg(GOF18-EGFP) cell line from (Greber et al., 2010)
Mouse: ESC_WT_E14 provided by Hans R. Schöler's lab
Mouse: ESC_IBc144_TetON-Esrrb produced in this paper
Mouse: ESC_Esrrb fl/fl-mKO2; MerCreMer the same cell line from (Adachi et al., 2018)
Mouse: ESC_beta-catenin fl/fl cell line from (Brault et al., 2001)
Mouse: ESC_TCF/Lef:H2B-GFP produced in this paper
Mouse: ESC_beta-catenin Exon-3 fl/fl Gift from Prof. Rolf Kemler
Mouse: ESC_IBc13_beta-catenin Exon-3 fl/fl; Cre-ERT2 produced in this paper
Mouse: ESC_IBc74_GSK_mutated_beta- catenin produced in this paper
Mouse: ESC_IBc19_beta-catenin fl/fl; Cre-ERT2 produced in this paper
Mouse: ESC_IBc76_beta-catenin fl/fl; Cre-ERT2;Eα-fusion produced in this paper
Mouse: ESC_IBc81_Nr0b1 produced in this paper
Mouse: ESC_IBc84_Tfcp2l1 produced in this paper
Mouse: ESC_IBc83_Nanog produced in this paper
Mouse: ESC_IBc85_Klf2 produced in this paper
Mouse: ESC_IBc82_Esrrb produced in this paper
Mouse: ESC_IBc139_Ntn1 produced in this paper
Mouse: ESC_IBc140_Krt18 produced in this paper
Mouse: ESC_IBC141_Arl4c produced in this paper
Mouse: ESC_IBC142_Spry2 produced in this paper
Mouse: ESC_IBC143_TetON-Spry2 produced in this paper
Mouse: ESC_IBC286_beta-catenin fl/fl; Cre-ERT2;Eα-fusion; Esrrb produced in this paper

Authentication

The genotype of all newly established cell lines were verified by PCR, Western blot or immunofluorescence. The results of these analysis are provided in the main and supplementary figures.

Mycoplasma contamination

These cell lines are negative for mycoplasma contamination:
Mouse: EpiSC_E3: Tg(GOF18-EGFP) cell line from (Greber et al., 2010)
Mouse: ESC_WT_E14 provided by Hans R. Schöler's lab
Mouse: ESC_Esrrb fl/fl-mKO2; MerCreMer the same cell line from (Adachi et al., 2018)
Mouse: ESC_TCF/Lef:H2B-GFP produced in this paper
Mouse: ESC_beta-catenin Exon-3 fl/fl Gift from Prof. Rolf Kemler
Mouse: ESC_IBc13_beta-catenin Exon-3 fl/fl; Cre-ERT2 produced in this paper
Mouse: ESC_IBc74_GSK_mutated_beta- catenin produced in this paper

These cell lines are not tested for mycoplasma contamination:
Mouse: ESC_IBc81_Nr0b1 produced in this paper
Mouse: ESC_IBc84_Tfcp2l1 produced in this paper
Mouse: ESC_IBc83_Nanog produced in this paper
Mouse: ESC_IBc85_Klf2 produced in this paper
Mouse: ESC_IBc82_Esrrb produced in this paper
Mouse: ESC_IBc139_Ntn1 produced in this paper
Mouse: ESC_IBc140_Krt18 produced in this paper
Mouse: ESC_IBC141_Arl4c produced in this paper
Mouse: ESC_IBC142_Spry2 produced in this paper
Mouse: ESC_IBC143_TetON-Spry2 produced in this paper
Mouse: ESC_IBc144_TetON-Esrrb produced in this paper
Mouse: ESC_IBc19_beta-catenin fl/fl; Cre-ERT2 produced from this paper
Mouse: ESC_IBc76_beta-catenin fl/fl; Cre-ERT2;Eα-fusion produced from this paper
Mouse: ESC_IBC286_beta-catenin fl/fl; Cre-ERT2;Eα-fusion; Esrrb produced from this paper

Commonly misidentified lines (See ICLAC register)	No commonly misidentified cell lines were used.

Animals and other organisms

Policy information about studies involving animals; ARRIVE guidelines recommended for reporting animal research

Laboratory animals	Mouse: WT C57BL/6 Bred in house<br>Mouse: WT CD1 Bred in house<br>Mouse: WT B6C3F1 Bred in house<br>Mouse: TCF/Lef:H2B-GFP (Ferrer-Vaquer et al., 2010)<br>Mouse: beta-catenin fl/fl (Brault et al., 2001)<br>Mouse: Esrrb fl/fl (Adachi et al., 2018)<br>Mouse: Wntless fl/fl (Carpenter et al., 2010)<br>Mouse: Zp3-Cre (Lewandoski et al., 1997)
Wild animals	No wild animals were used in this work.
Field-collected samples	No Field-collected samples were used in this work
Ethics oversight	Animal experiments and husbandry were performed according to the German Animal Welfare guidelines and approved by the Landesamt für Natur, Umwelt und Verbraucherschutz Nordrhein-Westfalen (State Agency for Nature, Environment and Consumer Protection of North Rhine-Westphalia)

Note that full information on the approval of the study protocol must also be provided in the manuscript.

Flow Cytometry

Plots

Confirm that:

- [x] The axis labels state the marker and fluorochrome used (e.g. CD4-FITC).
- [x] The axis scales are clearly visible. Include numbers along axes only for bottom left plot of group (a 'group' is an analysis of identical markers).
- [x] All plots are contour plots with outliers or pseudocolor plots.
- [x] A numerical value for number of cells or percentage (with statistics) is provided.

Methodology

Sample preparation	For sorting Venus positive cells, cells were dissociated using trypsin and transferred into PBS supplemented with 3% FCS.
Instrument	FACSAria IIu sorter (BD biosciences) was used for sorting and analysis.
Software	FACSDiva for sorting and FlowJo for analysis
Cell population abundance	After the sorting the cell population identity and abundance was verified by transgene expression using immunoflourescence staining.
Gating strategy	Single viable cells were first selected based on forward scatter area/side scatter area and forward scatter width/forward scatter area gating to select for live cells and then sorted for Venus expression.

- [x] Tick this box to confirm that a figure exemplifying the gating strategy is provided in the Supplementary Information.
