# Supplementary material for: Wnt/Beta-catenin/Esrrb signalling controls the tissue-scale reorganization and maintenance of the pluripotent lineage during murine embryonic diapause
Source: Nat Commun. 2020 Oct 30;11:5499. doi: 10.1038/s41467-020-19353-0 (PMC7603494; doi:10.1038/s41467-020-19353-0)

Figure S3b

Esrrb and Nanog overexpression

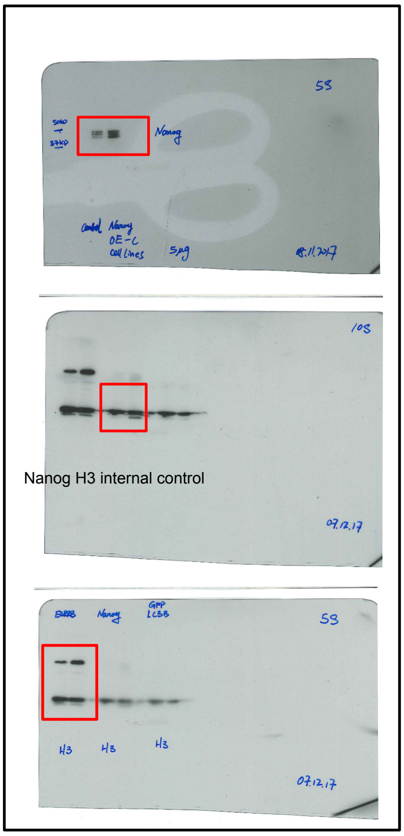

Figure 4a

Esrrb deletion

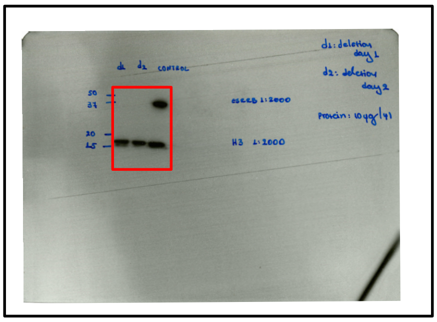

Figure S1c

Beta-catenin deletion

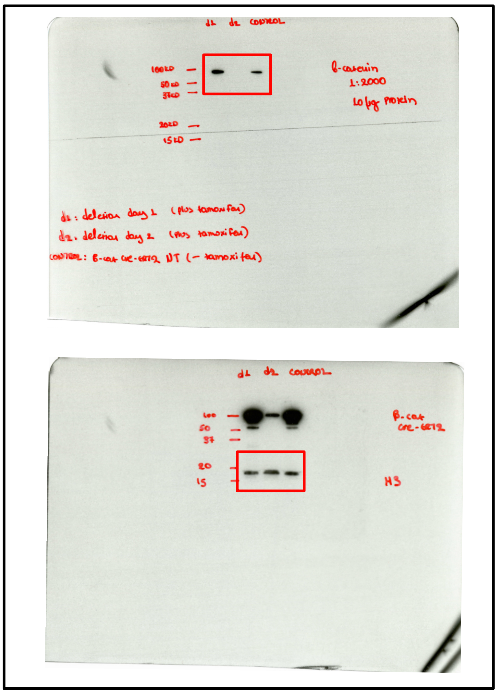

Figure S1a

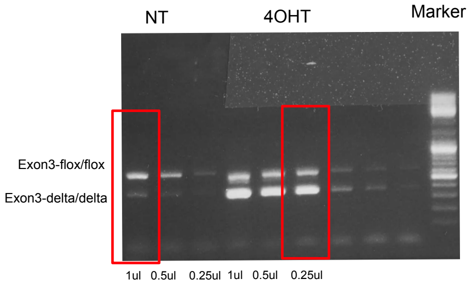

Figure S1b

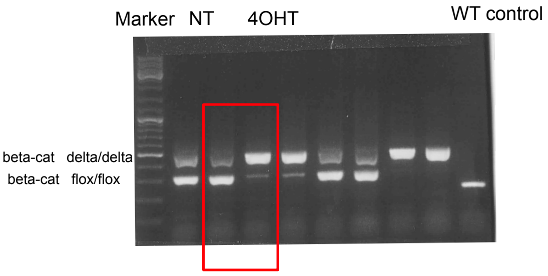

FACS plot for sorting Venus positive cells

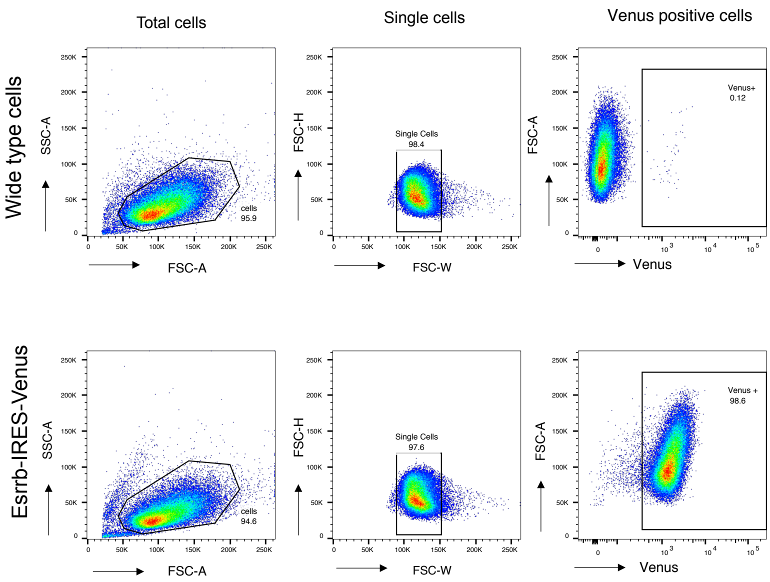

Supplement: Supplementary file 10 — Source Data [file 41467_2020_19353_MOESM10_ESM.zip › Gels Blots FACS.pdf]
